# CONS-COCOMAPS: a novel tool to measure and visualize the conservation of inter-residue contacts in multiple docking solutions

**DOI:** 10.1186/1471-2105-13-S4-S19

**Published:** 2012-03-28

**Authors:** Anna Vangone, Romina Oliva, Luigi Cavallo

**Affiliations:** 1 Department of Chemistry and Biology, University of Salerno, Via Ponte Don Melillo, Fisciano (SA), 84084, Italy; 2 Department of Applied Sciences, University "Parthenope" of Naples, Centro Direzionale Isola C4, Naples, 80143, Italy

## Abstract

**Background:**

The development of accurate protein-protein docking programs is making this kind of simulations an effective tool to predict the 3D structure and the surface of interaction between the molecular partners in macromolecular complexes. However, correctly scoring multiple docking solutions is still an open problem. As a consequence, the accurate and tedious screening of many docking models is usually required in the analysis step.

**Methods:**

All the programs under CONS-COCOMAPS have been written in python, taking advantage of python libraries such as SciPy and Matplotlib. CONS-COCOMAPS is freely available as a web tool at the URL:

http://www.molnac.unisa.it/BioTools/conscocomaps/.

**Results:**

Here we presented CONS-COCOMAPS, a novel tool to easily measure and visualize the consensus in multiple docking solutions. CONS-COCOMAPS uses the conservation of inter-residue contacts as an estimate of the similarity between different docking solutions. To visualize the conservation, CONS-COCOMAPS uses intermolecular contact maps.

**Conclusions:**

The application of CONS-COCOMAPS to test-cases taken from recent CAPRI rounds has shown that it is very efficient in highlighting even a very weak consensus that often is biologically meaningful.

## Background

Most important molecular processes in the cell rely on the interaction between biomolecules. Understanding the molecular basis of the recognition in a functional biological complex is thus a fundamental step for possible biomedical and biotechnological applications. However, the 3D structure of a significant fraction of biomolecular complexes is difficult to solve experimentally. In this scenario, the development of accurate protein-protein docking programs is making this kind of simulations an effective tool to predict the 3D structure and the surface of interaction between the molecular partners in macromolecular complexes [1]. Unfortunately, correctly scoring the obtained solutions to extract native-like ones is still an open problem [2,3], which is recently also object of assessment in CAPRI (Critical Assessment of PRedicted Interactions), a community-wide blind docking experiment [4]. As a consequence, the confidence to have a near-native solution among the ten best ranked ones is still an unreached task [3]. This requires the accurate and tedious screening of many docking models in the analysis step.

Typically, the first step of a docking simulation generates a large number, around 10^5^-10^6^, of 3D models (decoys). Such decoys are then clusterized on the basis of RMSD values, usually calculated on the atoms of the smaller molecular partner (or "ligand") [5-7]. The different solutions are ranked according to the cluster population: the most populated the cluster, the higher the rank. However, RMSD has two major limitations: i) its statistical significance is length dependent and ii) it is a global metric, that may not be able to characterize local similarities. As a consequence, solutions belonging to different RMSD-based clusters may share a notable number of intermolecular contacts, pointing essentially to the same interface. Therefore, as already reported [3,8,9], RMSD cannot be the only descriptor for the similarity of multiple docking solutions. Indeed, in the CAPRI experiment the correctness of a prediction, i.e. its similarity to the native structure, is assessed not only by means of RMSD based criteria, but also from the conservation of ligand-receptor contacts, as compared to the native structure [9]. Alternative scores have also been proposed to evaluate the correctness of a docking prediction, based on the geometric distance between the interfaces, and the residue-residue contact similarity [8].

However, the normal case in real-life research is having many different docking solutions to analyse and obviously no native structure to compare them to. Therefore, it would be of great utility both for bioinformaticians and wet biologists to have programs and tools to easily and effectively analyse and compare multiple docking solutions, based on criteria other than 'simple' RMSD. Most of all, it would be useful to visualize the consensus of multiple docking solutions, in order to appreciate at a glance which is the conservation rate of the predicted interface and which are the residues most often predicted as interacting.

As a matter of fact, if different docking solutions, especially from a series of well recognized programs, point to the same interacting regions, it is likely that the prediction can be better trusted. Consequently, it will be reasonable to focus attention, as for instance in site-directed mutagenesis experiments, on the residues most frequently predicted to be involved in the interaction. The concept of "consensus" has indeed been widely demonstrated to improve the performance of bioinformatics tools in many fields, including the prediction of protein and RNA secondary structure [10-16], of membrane protein topology [17], of protein retention in bacterial membrane [18], of docking small ligands to proteins [19,20], etc. Recently, consensus interface prediction has also been used to improve the performance of macromolecular docking simulations [21-23].

However, although many valuable tools have been made available to analyse the interface in biomolecular complexes [24-32], no tool has been developed to the aim of measuring and visualizing the consensus of multiple docking solutions. We recently developed COCOMAPS (bioCOmplexes COntact MAPS, available at the URL [33]), a comprehensive tool to analyse and visualize the interface in biological complexes, by making use of intermolecular contact maps [32]. We have shown that intermolecular contact maps can be very effective in providing an immediate 2D-view of the interaction, allowing to easily discriminate between similar and different binding solutions. They represent a sort of fingerprint of the complex, providing the crucial information in a ready-to-read form.

Here we use intermolecular contact maps as the basis for a novel tool, CONS-COCOMAPS (CONSensus-COCOMAPS), developed to measure and visualize the conservation of inter-residue contacts in multiple docking solutions. CONS-COCOMAPS provides both numerical values of the contacts conservation and a graphical representation in the form of a "consensus map". To show its performance, here we applied CONS-COCOMAPS to the analysis and visualization of a few test cases taken from recent CAPRI rounds.

## Methods

Given an ensemble of N models of the same biomolecular complex, the pairwise contacts conservation score, $C_{pair}^{ij}$, between models i and j is calculated as in Eq. 1.

(1) $$C_{pair}^{ij}=\frac{nc_{ij}}{\left(nc_{i}+nc_{j}\right)/2}$$

where *nc*_i _and *nc*_j _are the total number of inter-residue contacts in models i and j, respectively, and *nc*_ij _is the total number of inter-residue contacts common to models i and j. Following this definition, the average pairwise contacts conservation score $C_{pair}^{av}$ simply is the value of $C_{pair}^{ij}$ averaged over all the possible pairs of models in the considered ensemble, see Eq. 2.

(2) $$C_{pair}^{av}=\overset{N}{\underset{i,j>i}{\sum }}\frac{C_{pair}^{ij}}{N\left(N-1\right)/2}$$

However, Eq 1. can be generalized to a conservation score defined over all the N models in the considered ensemble, as in Eq.3.

(3) $$C_{100}=\frac{nc_{100}}{\overset{N}{\underset{i}{\sum }}\frac{nc_{i}}{N}}$$

where *nc*_100 _is the total number of inter-residue contacts common to all (100%) the models in the ensemble. The contacts conservation score of Eq. 3 can be extended to measure any amount of inter-residue contacts common to a given percentage of analysed models. For instance, C_70 _is calculated as in Eq. 4, where *nc*_70 _is the total number of inter-residue contacts conserved in 70% of the analysed models.

(4) $$C_{70}=\frac{nc_{70}}{\overset{N}{\underset{i}{\sum }}\frac{nc_{i}}{N}}$$

The total number of inter-residue contacts in an ensemble of N models, Nt, is calculated as in Eq. 5.

(5) $$Nt=\overset{N}{\underset{i}{\sum }}nc_{i}.$$

Finally, on a residue level we define the conservation rate, CR_kl_, of Eq. 6, where *nc*_kl _is the total number of models where residues k and l are in contact.

(6) $$CR_{kl}=\frac{nc_{kl}}{N}.$$

Within this work, two residues are defined in contact if any pair of atoms belonging to the two residues is closer than a cut-off distance of 5 Å, which is the threshold distance adopted in the assessment of CAPRI predictions to define native residue-residue contacts [9]. Conservation rates can be plotted in the form of consensus contact maps, which are depicted in a grey scale. The highest conservation corresponds to a black dot, absence of conservation corresponds to white, and contacts at increasing conservation appear in darker grey.

All the programs under CONS-COCOMAPS have been written in python, taking advantage of python libraries such as SciPy and Matplotlib. It is freely available as a web tool at the URL [34]).

### CAPRI models

The docking models for recent CAPRI targets were downloaded from the official web site (at the URL [35]). We selected seven recent protein-protein targets (T24-T26, T28-T29, T32, T36) for which the docking models were made available to the public. Four of them, T25, T26, T29 and T32, have at least one medium quality prediction and are more extensively discussed in the text. A total of 2130 CAPRI models have been analysed, 300 for target T24, round 9, 300 for target 25, round 9, 310 for target 26, round 10, 320 for target 28, round 12, 350 for target 29, round 13, 350 for target 32, round 15, and 200 for target 36, round 15 (see Table 1). Note that targets T24 and T25 refer to the same native complex. The quality score (Q-score) for each Predictor was calculated by summing 0, 1, 2 and 3 for each incorrect, acceptable, medium quality and high quality solution, respectively, as assessed in CAPRI [4]. Predictors which submitted less than the ten allowed models and those who submitted models with a ligand and/or receptor sequence not corresponding to the target were excluded from the analysis. L_rmsd is the pair-wise RMSD calculated on all the heavy atoms of the ligand after a LSQ RMS fit of the receptor invariant residues backbone, as in the CAPRI assessment [9].

**Table 1:** Analysed models

Target	CAPRI Round	Incorrect	Acceptable	Medium quality	High quality	All
T24	R 09	296	4	0	0	300
T25	R 09	268	19	12	1	300
T26	R 10	276	19	15	0	310
T28	R 12	320	0	0	0	320
T29	R 13	333	8	9	0	350
T32	R 15	316	6	13	15	350
T36	R 15	199	1	0	0	200

## Results and discussion

Given a number of multiple docking solutions, we calculated the conservation score of the inter-residue contacts at different percentages, from 0 to 100%. For instance, C_70 _gives the amount of inter-residue contacts which are conserved in 70% of the compared models. When only two models are compared, the pair-wise conservation score,$C_{pair}^{ij}$, is calculated. CONS-COCOMAPS then plots the inter-residue contacts conservation to an intermolecular contact map, that we call "consensus map".

The conservation of inter-residue contacts has been here measured and visualized with CONS-COCOMAPS for a total of 2130 models submitted to CAPRI for seven different targets: T24, T25, T26, T28, T29, T32 and T36 (See Table 1). The percentage of correct solutions among those submitted is 10-11% for T25, T26 and T32 and 5% for T29. For the remaining targets, T24, T28 and T36, it is instead much lower: 1% and 0% and 0.5%, respectively (see Table 1).

### Inter-residue conservation versus L_rmsd

**Figure 1:**
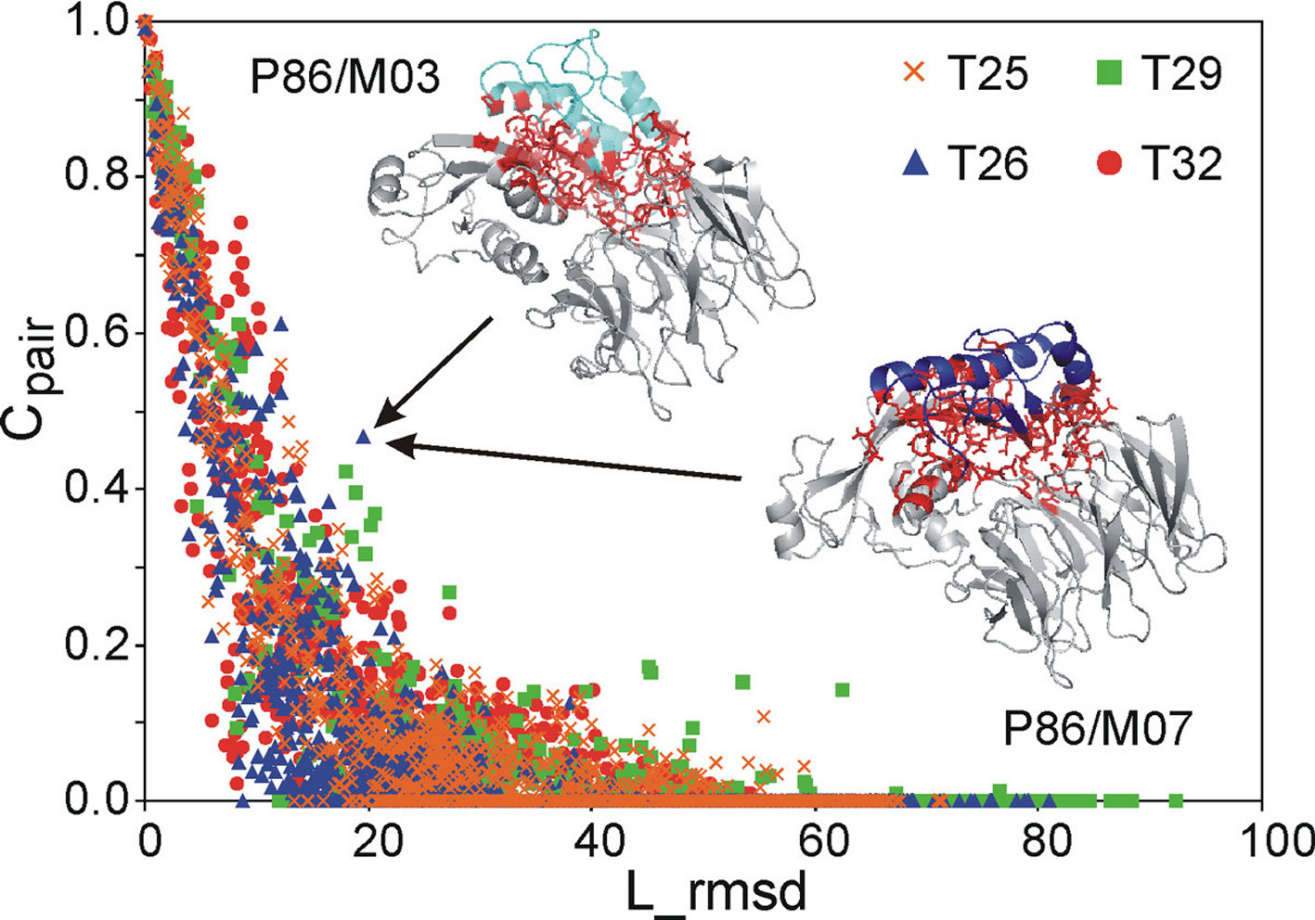
**$C_{pair}^{ij}$ versus L_rmsd**. Chart of the $C_{pair}^{ij}$ values versus L_rmsd values for targets T25, T26, T29 and T32. A comparison of the M03 and M07 models submitted by the P86 predictor for T26 and corresponding to the point indicated by the arrows is also shown with the ligand coloured in cyan and blue, respectively; residues involved in the contacts common to the two models are shown as red sticks.

The pair-wise conservation score, $C_{pair}^{ij}$, between all the models within each of the CAPRI targets T25, T26, T29 and T32 have been plotted versus the corresponding L_rmsd values in Figure 1. As expected, $C_{pair}^{ij}$ rapidly decreases as the L_rmsd increases, with $C_{pair}^{ij}$ approaching to zero at L_rmsd higher than 30-40 Å. The $C_{pair}^{ij}$ distribution is significantly spread out, even at $C_{pair}^{ij}$ values around 0.5 (which means that one out of two contacts at the interface is conserved in the two considered models), and several outliers are indeed observed that contemporarily show either low $C_{pair}^{ij}$ and low L_rmsd values or high $C_{pair}^{ij}$ and high L_rmsd values. As an example, the 3D representation of the models M03 and M07 submitted by the P86 predictor for T26, responsible for the point outlined by the arrows, is shown in the same Figure. The L_rmsd for their superimposition is as high as 19.6 Å, notwithstanding a pair-wise conservation score $C_{pair}^{ij}$ of 0.47 is calculated. This is due to a significant conformational change undergone by both the receptor and the ligand in the two models (RMSD for the best superposition of the two receptors and the two ligands is 4.8 Å and 2.8 Å, respectively), which causes a remarkably different orientation of the ligand. Nevertheless, regions involved in the interaction are substantially the same, because the ligand somehow "follows" the receptor in its conformational change. This case and many others demonstrate once more that the RMSD cannot be selected as the only descriptors for the similarity of two docking solutions and that descriptors directly describing the property of interest, in this case the interface, should be used [3,8,9].

### Conservation and Consensus maps for the multiple solutions submitted by each predictor

Conservation scores have also been calculated for each set of ten models submitted for each CAPRI target by the same predictor. C_30_, C_50 _and C_70 _are reported in the Additional file 1. They correspond to the amount of inter-residue contacts which are conserved in 30%, 50% and 70% of the models, respectively. The average $C_{pair}^{av}$ and the quality score, Q-score, for each predictor, obtained on the basis of the CAPRI assessment, are also reported.

**Figure 2:**
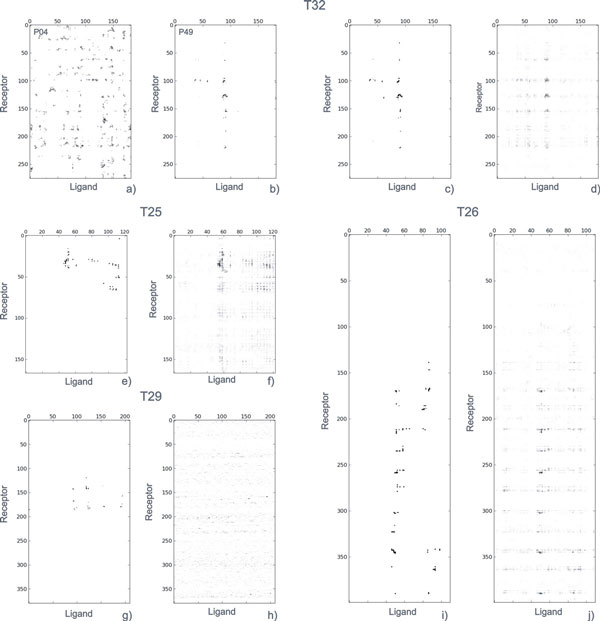
**Consensus maps. (a-b) **CONS-COCOMAPS consensus maps obtained from the 10 models submitted for the CAPRI target T32 by the P04 and P49 predictors. **c-j) **Comparison between the CONS-COCOMAPS consensus maps **(d,f,h,j) **obtained from all the 300, 310, 350 and 350 models submitted to CAPRI for the targets T25, T26, T29 and T32, respectively, and the intermolecular contact maps **(c,e,g,i) **of the corresponding native structures (PDB codes: 2J59, 2HQS, 2VDU and 3BX1).

As expected, the inter-residue conservation rate within each set of multiple solutions submitted by each predictor is very variable. As an illustrative example, in Figure 2a-b, the graphical CONS-COCOMAPS outputs (consensus maps) are shown for the set of ten predictions submitted by predictors P04 and P49 for target T32. For comparison, the intermolecular contact map for the native structure (PDB code 3BX1, [36]) is also reported (Figure 2c). The calculated $C_{pair}^{av}$ values are 0.003 and 0.400 for predictors P04 and P49, respectively. Visual inspection of Figure 2a-b immediately indicates that the solutions proposed by predictor P49 are very conservative as concerns the predicted inter-residue contacts, whereas the predicted inter-residue contacts in the solutions proposed by predictor P04 are extremely diverse and spread out all over the map. Further, the maps of Figure 2b-c also immediately show that the consensus contact map of predictor P49 is extremely similar to the contact map of the native complex structure. In fact, predictor P49 performed very well in this test case, having one acceptable, two medium quality and five high quality predictions. On the contrary, predictor P04 had only incorrect predictions.

We noted that there is indeed a nice correlation, especially for targets T26 and T32, between the success of the predictor and a high conservation of the inter-residue contacts. However, it is worth to remark that the opposite does not hold true, i.e. we also observed cases where a predictor submitted very similar predictions in terms of inter-residue contacts but they were far away from the native structure. For instance, the ten predictions submitted by predictor P89 for target T25 share an average $C_{pair}^{av}$ as high as 0.772, notwithstanding all the predictions have been assessed as incorrect. The corresponding consensus map is shown and compared with the native structure contact map in the Additional file 2.

### Consensus maps for the multiple solutions submitted by all the predictors

Overall conservation scores of the inter-residue contacts in all the models submitted for the analysed targets are quite low. Conservation scores at 5, 10, 15 and 20% are reported in Table 2 both for all the docking models and for only the incorrect solutions. They correspond to the number of inter-residue contacts which are conserved in 5, 10, 15 and 20 models out of 100, divided by the average number of contacts per model. From Table 2 it is apparent that the conservation of inter-residue contacts in T24, T28, T29 and T36 is particularly low. The conservation score of contacts common to the 5% of all the models, including the correct ones, is indeed below 0.7 (0.398, 0.056, 0.176 and 0.643, respectively). At higher percentages the conservation scores for these targets are zero, with the only exception of T36, whose C10 value is 0.016.

**Table 2:** Inter-residue conservation scores at different percentages for all the models submitted for each target

Target	Nt	C5	C10	C15	C20
T24	15818	0.398	0.000	0.000	0.000
T24-incorrect^a^	15618	0.322	0.000	0.000	0.000

T25	15399	2.455	0.448	0.078	0.000
T25-incorrect^a^	13613	1.477	0.020	0.000	0.000

T26	22063	2.318	0.576	0.183	0.020
T26-incorrect^a^	19825	2.019	0.125	0.014	0.000

T28	29360	0.056	0.000	0.000	0.000

T29	23890	0.176	0.000	0.000	0.000
T29-incorrect^a^	22923	0.000	0.000	0.000	0.000

T32	25859	2.274	0.420	0.081	0.027
T32-incorrect^a^	23420	1.754	0.202	0.027	0.000

T36	12750	0.643	0.016	0.000	0.000
T36-incorrect^a^	12673	0.628	0.016	0.000	0.000

^a ^Calculations performed upon excluding all the correct predictions.

On the contrary, C5 assumes higher and similar values for the other three targets, from 2.274 for target T32 to 2.455 for target T25. These values are remarkably lower when the correct predictions are excluded from the analysis. C10 values are also quite similar and range from the 0.420 for target T32 to 0.576 for target T26. C15 values are more variable, ranging from 0.078 for target T25 to 0.183 for target T26. Exclusion of the correct predictions causes a dramatic decrease of the C15 values, which approach to zero. At percentages of 20% or more, the conservation score is not higher than 0.027 for any of the analysed targets.

Conservation rates at the residue level have been plotted in consensus maps and are reported in Figure 2 for T25, T26, T29 and T32 and in the Additional file 3 for T24, T28 and T36, together with the intermolecular contact map of the corresponding native structures (PDB codes: 2J59[37], 2HQS[38], 2ONI, 2VDU[39], 3BX1 [36] and 2W5F[40] for T24/T25, T26, T28, T29, T32 and T36, respectively). The consensus maps reported in Figures 2d, f, h, j and 2S_b,d,f _therefore represent the consensus emerging from the analysis of 200 to 350 different solutions, for each target, submitted by different predictors and obtained and selected on the basis of different methods and criteria.

As a consequence of their very low conservation scores, the consensus maps of T24, T28, T29 and T36 are quite spread out and only for T24 a week signal emerges from the background noise (Figures 2h and 2Sb_,d,f_). On the contrary, in case of targets T25, T26 and T32, some darker hot spots, due to the best conserved inter-residue contacts in the multiple solutions, clearly emerge (Figure 2b, d, f,). Interestingly, analysis of the CONS-COCOMAPS outputs indicates that among the ten inter-residue contacts with highest conservation rates, reported in Table 3 several correspond to native inter-residue contacts. Indeed, for targets T25, T26 and T32, seven, nine and eight of the ten most conserved contacts correspond to distances within 5 Å in the native structure [36-39] (see again Table 3). Considering that only ~10% of the CAPRI models for the three targets was assessed to be correct (Table 1), this indicates that focusing on the consensus of predicted inter-residue contacts, rather than on the correctness of the entire models, can significantly increase the success rate of the prediction. Importantly, hot spots of the interactions are highlighted by this approach, such as for instance residue Tyr87 of the T32 ligand (the barley α-amylase/subtilisin inhibitor), whose mutation to alanine has been experimentally shown to dramatically decrease the ligand-receptor affinity [36]. A useful consensus, five correct contacts among the ten most conserved contacts, also emerges for T29, for which only 5% of the models was assessed to be correct (Table 3). Further, when drawing the consensus maps for targets T25, T26 and T32 using only the incorrect solutions, some inter-residue contacts corresponding to the native ones still emerge, and are clearly distinguishable from the noise (Additional file 4). In particular, considering only the incorrect models submitted for T25, T26 and T32, two, seven and four contacts, respectively, correspond to native ones (data not shown). Surprisingly, even T24, having no medium/high quality prediction, presents three native contacts among the ten most conserved ones (Additional file 5). Quite strikingly, these findings indicate that the consensus of many solutions, even incorrect according to the CAPRI definition, may point to the correct inter-residue contacts. If confirmed, this result could be of great interest and utility in applications such as mutagenesis experiments design, considering that the main aim of bioinformaticians and wet biologists, when performing macromolecular docking simulations, is often to predict the residues at the interface, more than the fine details of the biomolecular complex.

**Table 3:** Ten most conserved inter-residue contacts.

	CR_kl_	Receptor		Ligand		Distance (Å)
**T25**						
	0,173	TYR	35	TYR	999	3,48
	0,167	PHE	51	ASP	996	**5,82**
	0,163	PHE	51	ILE	1053	4,00
	0,150	ASN	52	ASP	996	3,84
	0,147	THR	44	TYR	999	2,60
	0,140	ASN	52	TYR	999	4,20
	0,140	ILE	46	ILE	997	3,65
	0,137	THR	45	TYR	999	3,49
	0,133	ILE	49	GLN	1035	**6,09**
	0,130	ILE	49	ILE	995	**5,29**

**T26**						
	0,232	GLU	293	GLU	116	3,62
	0,210	GLU	293	THR	114	2,66
	0,197	PHE	424	PRO	115	3,43
	0,190	ALA	249	GLU	116	2,92
	0,187	SER	205	GLU	116	2,66
	0,174	PHE	424	GLU	116	**5,55**
	0,174	HIS	246	GLU	116	2,79
	0,168	MET	204	GLU	116	3,75
	0,158	GLN	336	THR	114	2,94
	0,158	GLY	248	GLU	116	3,94

**T29**						
	0,069	TRP	236	PHE	165	**7,67**
	0,063	HIS	221	PHE	165	3,65
	0,063	VAL	195	ARG	195	**6,53**
	0,060	TRP	236	GLU	204	3,03
	0,057	PHE	231	PRO	236	3,88
	0,057	LYS	223	THR	200	**5,73**
	0,054	VAL	195	PHE	165	**7,28**
	0,051	PHE	231	LEU	237	3,35
	0,051	TRP	236	TYR	207	3,67
	0,051	VAL	233	THR	200	**6,82**

**T32**						
	0,223	LEU	126	TYR	87	3,71
	0,200	GLY	127	TYR	87	3,74
	0,183	SER	125	TYR	87	**7,68**
	0,169	GLY	100	TYR	87	4,03
	0,160	ASN	62	TYR	87	**9,91**
	0,157	SER	128	TYR	87	3,49
	0,146	ASN	62	THR	89	4,65
	0,143	ASN	155	THR	89	4,56
	0,140	LEU	96	TYR	87	3,52
	0,137	GLY	127	LEU	91	3,51

The ten most conserved inter-residue contacts are reported for targets T25, T26, T29 and T32, together with corresponding distances in the native structures [36-39]. Distances above 5 Å are outlined in bold.

## Conclusions

Here we presented CONS-COCOMAPS, a novel tool to easily measure and visualize the consensus in multiple docking solutions. CONS-COCOMAPS uses the conservation of inter-residue contacts as an estimate of the similarity between different docking solutions. The conservation of ligand-receptor contacts is indeed used as one of the fundamental criteria in CAPRI for assessing the similarity of a predicted complex to the native structure, and recently it has been emphasized that it can be the most useful descriptor when looking at the biological significance of the prediction, i.e. the individuation of the interface area [3]. To visualize the conservation, CONS-COCOMAPS uses intermolecular contact maps, that we recently showed to be a very effective way to visualize a biomolecular complex interface [32]. There is virtually no limit on the number of models that can be compared by CONS-COCOMAPS. This novel tool is freely available to the scientific community (at the URL [34]) and can straightforwardly be applied to the analysis of the outputs of one or more docking programs.

The application of CONS-COCOMAPS to some test-cases taken from recent CAPRI rounds shows that it is efficient in highlighting even a very weak consensus. Interestingly, in three out of the seven analysed cases, T25, T26 and T32, consensus maps clearly point to the native contacts (Figure 2 and Table 3). In other two cases, T24 and T29, although the consensus is less visually apparent from the maps (Figure 2 and Additional file 3), three and five native contacts, respectively, are included among the ten most conserved inter-residue contacts (Table 3 and Additional file 5). Importantly, in none of the analysed cases a false-positive consensus emerged. This opens the road to further studies to test and prove whether the consensus of a large number of docking solutions may be used to successfully predict residue-residue contacts in biomolecular complexes.

## Competing interests

The authors declare that they have no competing interests.

## Authors' contributions

AV carried out the measures, wrote the code, implemented the web server and helped to draft the manuscript. RO and LC conceived of the study, and participated in its design and coordination and drafted the manuscript. All authors read and approved the final manuscript.

## Supplementary Material

Additional file 1 **Inter-residue conservation scores**. Table reporting inter-residue conservation scores at different percentages of the ten docking solutions submitted to CAPRI by each Predictor. The Q-score, based on the CAPRI assessment, is also reported for each Target/Predictor. Click here for file

Additional file 2 **Consensus map from the P89 predictor for T25**. Comparison between the CONS-COCOMAPS consensus map (b) obtained from the 10 models submitted for the CAPRI target T25 by the P89 predictor, and the intermolecular contact map (a) of the corresponding native structure (PDB code: 2J59). Click here for file

Additional file 3 **Consensus maps for T24, T28 and T36**. Comparison between the CONS-COCOMAPS consensus maps (b,d,f) obtained from all the 300, 320 and 200 models submitted to CAPRI for the targets T24, T28 and T36, respectively, and the intermolecular contact maps (a,c,e) of the corresponding native structures (PDB codes: 2J59, 2ONI and 2W5F). Click here for file

Additional file 4 **Consensus maps for T25, T26 and T32 from incorrect models**. Comparison between the CONS-COCOMAPS consensus maps (b,d,f) obtained from the 268, 276 and 316 incorrect models submitted to CAPRI for the targets T25, T26 and T32, respectively, and the intermolecular contact maps (a,c,e) of the corresponding native structures (PDB codes: 2J59, 2HQS and 3BX1). Click here for file

Additional file 5 **Ten most conserved inter-residue contacts for T24 and corresponding distances in the native structure**. Click here for file

## References

[B1] Janin J Protein-protein docking tested in blind predictions: the CAPRI experiment Mol Biosyst 2010 6 12 2351 2362 10.1039/c005060c 20725658

[B2] Bernauer J Aze J Janin J Poupon A A new protein-protein docking scoring function based on interface residue properties Bioinformatics 2007 23 5 555 562 10.1093/bioinformatics/btl654 17237048

[B3] Bourquard T Bernauer J Aze J Poupon A A collaborative filtering approach for protein-protein docking scoring functions PLoS One 6 4 e18541 21526112 10.1371/journal.pone.0018541 PMC3081294

[B4] Lensink MF Mendez R Wodak SJ Docking and scoring protein complexes: CAPRI Proteins 2007 69 3 4 704 718 10.1002/prot.21804 17918726

[B5] Comeau SR Gatchell DW Vajda S Camacho CJ ClusPro: an automated docking and discrimination method for the prediction of protein complexes Bioinformatics 2004 20 1 45 50 10.1093/bioinformatics/btg371 14693807

[B6] Gray JJ Moughon S Wang C Schueler-Furman O Kuhlman B Rohl CA Baker D Protein-protein docking with simultaneous optimization of rigid-body displacement and side-chain conformations J Mol Biol 2003 331 1 281 299 10.1016/S0022-2836(03)00670-3 12875852

[B7] de Vries SJ van Dijk AD Krzeminski M van Dijk M Thureau A Hsu V Wassenaar T Bonvin AM HADDOCK versus HADDOCK: new features and performance of HADDOCK2.0 on the CAPRI targets Proteins 2007 69 4 726 733 10.1002/prot.21723 17803234

[B8] Gao M Skolnick J New benchmark metrics for protein-protein docking methods Proteins 79 5 1623 1634 21365685 10.1002/prot.22987 PMC3076516

[B9] Mendez R Leplae R De Maria L Wodak SJ Assessment of blind predictions of protein-protein interactions: current status of docking methods Proteins 2003 52 1 51 67 10.1002/prot.10393 12784368

[B10] Pollastri G Martin AJ Mooney C Vullo A Accurate prediction of protein secondary structure and solvent accessibility by consensus combiners of sequence and structure information BMC Bioinformatics 2007 8 201 10.1186/1471-2105-8-201 17570843 PMC1913928

[B11] Albrecht M Tosatto SC Lengauer T Valle G Simple consensus procedures are effective and sufficient in secondary structure prediction Protein Eng 2003 16 7 459 462 10.1093/protein/gzg063 12915722

[B12] Colloc'h N Etchebest C Thoreau E Henrissat B Mornon JP Comparison of three algorithms for the assignment of secondary structure in proteins: the advantages of a consensus assignment Protein Eng 1993 6 4 377 382 10.1093/protein/6.4.377 8332595

[B13] Konings DA Hogeweg P Pattern analysis of RNA secondary structure similarity and consensus of minimal-energy folding J Mol Biol 1989 207 3 597 614 10.1016/0022-2836(89)90468-3 2474658

[B14] Kiryu H Kin T Asai K Robust prediction of consensus secondary structures using averaged base pairing probability matrices Bioinformatics 2007 23 4 434 441 10.1093/bioinformatics/btl636 17182698

[B15] Witwer C Hofacker IL Stadler PF Prediction of consensus RNA secondary structures including pseudoknots IEEE/ACM Trans Comput Biol Bioinform 2004 1 2 66 77 10.1109/TCBB.2004.22 17048382

[B16] Anwar M Nguyen T Turcotte M Identification of consensus RNA secondary structures using suffix arrays BMC Bioinformatics 2006 7 244 10.1186/1471-2105-7-244 16677380 PMC1475642

[B17] Bernsel A Viklund H Hennerdal A Elofsson A TOPCONS: consensus prediction of membrane protein topology Nucleic Acids Res 2009 37 Web Server W465 W468 19429891 10.1093/nar/gkp363 PMC2703981

[B18] Tjalsma H van Dijl JM Proteomics-based consensus prediction of protein retention in a bacterial membrane Proteomics 2005 5 17 4472 4482 10.1002/pmic.200402080 16220534

[B19] Ginalski K Rychlewski L Protein structure prediction of CASP5 comparative modeling and fold recognition targets using consensus alignment approach and 3D assessment Proteins 2003 53 Suppl 6 410 417 14579329 10.1002/prot.10548

[B20] Plewczynski D Lazniewski M von Grotthuss M Rychlewski L Ginalski K VoteDock: consensus docking method for prediction of protein-ligand interactions J Comput Chem 32 4 568 581 20812324 10.1002/jcc.21642 PMC4510457

[B21] de Vries SJ Bonvin AM CPORT: a consensus interface predictor and its performance in prediction-driven docking with HADDOCK PLoS One 6 3 e17695 21464987 10.1371/journal.pone.0017695 PMC3064578

[B22] Huang B Schroeder M Using protein binding site prediction to improve protein docking Gene 2008 422 1-2 14 21 10.1016/j.gene.2008.06.014 18616991

[B23] Qin S Zhou HX meta-PPISP: a meta web server for protein-protein interaction site prediction Bioinformatics 2007 23 24 3386 3387 10.1093/bioinformatics/btm434 17895276

[B24] Fischer TB Holmes JB Miller IR Parsons JR Tung L Hu JC Tsai J Assessing methods for identifying pair-wise atomic contacts across binding interfaces J Struct Biol 2006 153 2 103 112 10.1016/j.jsb.2005.11.005 16377205

[B25] Gabdoulline RR Wade RC Walther D MolSurfer: a macromolecular interface navigator Nucleic Acids Res 2003 31 13 3349 3351 10.1093/nar/gkg588 12824324 PMC168994

[B26] Kleinjung J Fraternali F POPSCOMP: an automated interaction analysis of biomolecular complexes Nucleic Acids Res 2005 33 Web Server W342 W346 15980485 10.1093/nar/gki369 PMC1160130

[B27] Cavallo L Kleinjung J Fraternali F POPS: a fast algorithm for solvent accessible surface areas at atomic and residue level Nucleic Acids Res 2003 31 13 3364 3366 10.1093/nar/gkg601 12824328 PMC169007

[B28] Krissinel E Henrick K Inference of macromolecular assemblies from crystalline state J Mol Biol 2007 372 3 774 797 10.1016/j.jmb.2007.05.022 17681537

[B29] Reynolds C Damerell D Jones S ProtorP: a protein-protein interaction analysis server Bioinformatics 2009 25 3 413 414 10.1093/bioinformatics/btn584 19001476

[B30] Salerno WJ Seaver SM Armstrong BR Radhakrishnan I MONSTER: inferring non-covalent interactions in macromolecular structures from atomic coordinate data Nucleic Acids Res 2004 32 Web Server W566 W568 15215451 10.1093/nar/gkh434 PMC441572

[B31] Tina KG Bhadra R Srinivasan N PIC: Protein Interactions Calculator Nucleic Acids Res 2007 35 Web Server W473 W476 17584791 10.1093/nar/gkm423 PMC1933215

[B32] Vangone A Spinelli R Scarano V Cavallo L Oliva R COCOMAPS: a web application to analyse and visualize contacts at the interface of biomolecular complexes Bioinformatics 2011 27 20 2915 2916 10.1093/bioinformatics/btr484 21873642

[B33] The CoCoMAPS Web Tool http://www.molnac.unisa.it/BioTools/cocomaps/

[B34] The CONS-COCOMAPS Web Tool http://www.molnac.unisa.it/BioTools/conscocomaps/

[B35] The CAPRI Official Web Site http://www.ebi.ac.uk/msd-srv/capri/

[B36] Micheelsen PO Vevodova J De Maria L Ostergaard PR Friis EP Wilson K Skjot M Structural and mutational analyses of the interaction between the barley alpha-amylase/subtilisin inhibitor and the subtilisin savinase reveal a novel mode of inhibition J Mol Biol 2008 380 4 681 690 10.1016/j.jmb.2008.05.034 18556023

[B37] Menetrey J Perderiset M Cicolari J Dubois T Elkhatib N El Khadali F Franco M Chavrier P Houdusse A Structural basis for ARF1-mediated recruitment of ARHGAP21 to Golgi membranes Embo J 2007 26 7 1953 1962 10.1038/sj.emboj.7601634 17347647 PMC1847662

[B38] Bonsor DA Grishkovskaya I Dodson EJ Kleanthous C Molecular mimicry enables competitive recruitment by a natively disordered protein J Am Chem Soc 2007 129 15 4800 4807 10.1021/ja070153n 17375930

[B39] Leulliot N Chaillet M Durand D Ulryck N Blondeau K van Tilbeurgh H Structure of the yeast tRNA m7G methylation complex Structure 2008 16 1 52 61 10.1016/j.str.2007.10.025 18184583

[B40] Najmudin S Pinheiro BA Prates JA Gilbert HJ Romao MJ Fontes CM Putting an N-terminal end to the Clostridium thermocellum xylanase Xyn10B story: crystal structure of the CBM22-1-GH10 modules complexed with xylohexaose J Struct Biol 172 3 353 362 20682344 10.1016/j.jsb.2010.07.009

