# Supplementary material for: CONS-COCOMAPS: a novel tool to measure and visualize the conservation of inter-residue contacts in multiple docking solutions
Source: BMC Bioinformatics. 2012 Mar 28;13(Suppl 4):S19. doi: 10.1186/1471-2105-13-S4-S19 (PMC3434444; doi:10.1186/1471-2105-13-S4-S19)
Supplement: Additional file 1 — Inter-residue conservation scores. Table reporting inter-residue conservation scores at different percentages of the ten docking solutions submitted to CAPRI by each Predictor. The Q-score, based on the CAPRI assessment, is also reported for each Target/Predictor. [file 1471-2105-13-S4-S19-S1.doc]

| **Target/Predictor** | **Nt** | **C_30_** | **C_50_** | **C_70_** |  | **Q-Score** |
| --- | --- | --- | --- | --- | --- | --- |
| T24/P02 | 660 | 0,061 | 0,000 | 0,000 | 0,020 | 0 |
| T24/P06 | 498 | 0,522 | 0,040 | 0,000 | 0,085 | 0 |
| T24/P07 | 305 | 0,590 | 0,164 | 0,066 | 0,121 | 0 |
| T24/P16 | 500 | 0,380 | 0,040 | 0,000 | 0,075 | 0 |
| T24/P20 | 817 | 0,012 | 0,000 | 0,000 | 0,031 | 0 |
| T24/P22 | 431 | 0,000 | 0,000 | 0,000 | 0,023 | 0 |
| T24/P23 | 383 | 0,104 | 0,000 | 0,000 | 0,027 | 0 |
| T24/P24 | 322 | 0,528 | 0,031 | 0,000 | 0,073 | 1 |
| T24/P30 | 349 | 0,000 | 0,000 | 0,000 | 0,011 | 0 |
| T24/P31 | 624 | 0,272 | 0,000 | 0,000 | 0,047 | 0 |
| T24/P32 | 464 | 0,216 | 0,000 | 0,000 | 0,036 | 0 |
| T24/P34 | 359 | 0,056 | 0,000 | 0,000 | 0,015 | 0 |
| T24/P39 | 641 | 0,374 | 0,031 | 0,000 | 0,045 | 0 |
| T24/P47 | 528 | 0,322 | 0,000 | 0,000 | 0,043 | 2 |
| T24/P50 | 394 | 0,102 | 0,000 | 0,000 | 0,021 | 0 |
| T24/P52 | 520 | 1,577 | 0,442 | 0,288 | 0,360 | 0 |
| T24/P54 | 490 | 0,816 | 0,082 | 0,020 | 0,116 | 0 |
| T24/P61 | 506 | 0,870 | 0,277 | 0,020 | 0,149 | 0 |
| T24/P63 | 404 | 0,173 | 0,000 | 0,000 | 0,032 | 0 |
| T24/P65 | 558 | 0,538 | 0,072 | 0,000 | 0,092 | 0 |
| T24/P69 | 615 | 1,138 | 0,146 | 0,000 | 0,152 | 0 |
| T24/P70 | 510 | 0,059 | 0,000 | 0,000 | 0,019 | 0 |
| T24/P72 | 413 | 0,073 | 0,000 | 0,000 | 0,024 | 0 |
| T24/P74 | 646 | 0,372 | 0,015 | 0,000 | 0,050 | 0 |
| T24/P81 | 651 | 0,077 | 0,000 | 0,000 | 0,023 | 1 |
| T24/P83 | 983 | 0,142 | 0,000 | 0,000 | 0,047 | 0 |
| T24/P89 | 522 | 0,038 | 0,000 | 0,000 | 0,023 | 0 |
| T24/P90 | 1108 | 0,135 | 0,000 | 0,000 | 0,032 | 0 |
| T24/P92 | 175 | 0,914 | 0,400 | 0,000 | 0,171 | 0 |
| T24/P94 | 442 | 1,787 | 0,769 | 0,000 | 0,353 | 0 |
| T25/P02 | 578 | 0,796 | 0,052 | 0,000 | 0,102 | 3 |
| T25/P06 | 549 | 0,820 | 0,273 | 0,036 | 0,141 | 4 |
| T25/P07 | 471 | 0,021 | 0,000 | 0,000 | 0,020 | 0 |
| T25/P08 | 339 | 0,649 | 0,059 | 0,000 | 0,080 | 0 |
| T25/P12 | 545 | 1,266 | 0,495 | 0,000 | 0,222 | 2 |
| T25/P16 | 465 | 0,086 | 0,000 | 0,000 | 0,022 | 2 |
| T25/P18 | 560 | 0,357 | 0,000 | 0,000 | 0,062 | 3 |
| T25/P20 | 692 | 0,014 | 0,000 | 0,000 | 0,016 | 2 |
| T25/P22 | 411 | 0,000 | 0,000 | 0,000 | 0,005 | 0 |
| T25/P23 | 442 | 0,860 | 0,113 | 0,000 | 0,122 | 5 |
| T25/P24 | 349 | 0,917 | 0,172 | 0,000 | 0,126 | 3 |
| T25/P30 | 424 | 0,142 | 0,000 | 0,000 | 0,054 | 3 |
| T25/P32 | 523 | 0,631 | 0,019 | 0,000 | 0,077 | 0 |
| T25/P33 | 497 | 0,000 | 0,000 | 0,000 | 0,018 | 0 |
| T25/P34 | 349 | 0,000 | 0,000 | 0,000 | 0,028 | 0 |
| T25/P39 | 478 | 0,251 | 0,000 | 0,000 | 0,041 | 0 |
| T25/P47 | 655 | 0,229 | 0,000 | 0,000 | 0,062 | 1 |
| T25/P52 | 291 | 1,375 | 0,584 | 0,000 | 0,217 | 0 |
| T25/P54 | 595 | 1,294 | 0,555 | 0,235 | 0,275 | 6 |
| T25/P61 | 697 | 0,316 | 0,000 | 0,000 | 0,038 | 2 |
| T25/P63 | 433 | 0,393 | 0,023 | 0,000 | 0,065 | 3 |
| T25/P65 | 547 | 0,055 | 0,000 | 0,000 | 0,017 | 0 |
| T25/P69 | 572 | 0,490 | 0,017 | 0,000 | 0,071 | 0 |
| T25/P70 | 496 | 0,060 | 0,000 | 0,000 | 0,027 | 2 |
| T25/P72 | 391 | 0,000 | 0,000 | 0,000 | 0,027 | 0 |
| T25/P74 | 370 | 1,189 | 0,081 | 0,054 | 0,144 | 0 |
| T25/P81 | 654 | 0,336 | 0,000 | 0,000 | 0,051 | 4 |
| T25/P83 | 925 | 0,032 | 0,000 | 0,000 | 0,021 | 0 |
| T25/P89 | 515 | 1,282 | 1,029 | 0,835 | 0,772 | 0 |
| T25/P90 | 586 | 0,051 | 0,000 | 0,000 | 0,023 | 1 |
| T26/P02 | 563 | 0,053 | 0,000 | 0,000 | 0,015 | 0 |
| T26/P04 | 949 | 0,000 | 0,000 | 0,000 | 0,007 | 0 |
| T26/P05 | 540 | 0,000 | 0,000 | 0,000 | 0,006 | 0 |
| T26/P06 | 1107 | 0,470 | 0,000 | 0,000 | 0,067 | 1 |
| T26/P17 | 493 | 0,000 | 0,000 | 0,000 | 0,009 | 0 |
| T26/P23 | 618 | 0,210 | 0,000 | 0,000 | 0,041 | 0 |
| T26/P25 | 489 | 0,736 | 0,164 | 0,000 | 0,110 | 3 |
| T26/P26 | 765 | 1,320 | 0,562 | 0,261 | 0,313 | 9 |
| T26/P27 | 468 | 0,000 | 0,000 | 0,000 | 0,008 | 0 |
| T26/P36 | 751 | 0,080 | 0,000 | 0,000 | 0,024 | 2 |
| T26/P37 | 525 | 0,457 | 0,000 | 0,000 | 0,046 | 0 |
| T26/P38 | 604 | 0,646 | 0,066 | 0,000 | 0,088 | 5 |
| T26/P39 | 1021 | 0,872 | 0,470 | 0,000 | 0,173 | 0 |
| T26/P40 | 804 | 0,734 | 0,075 | 0,000 | 0,115 | 1 |
| T26/P41 | 670 | 0,448 | 0,015 | 0,000 | 0,055 | 2 |
| T26/P45 | 588 | 0,102 | 0,000 | 0,000 | 0,028 | 0 |
| T26/P47 | 1009 | 1,100 | 0,218 | 0,010 | 0,163 | 1 |
| T26/P51 | 559 | 0,072 | 0,000 | 0,000 | 0,021 | 1 |
| T26/P55 | 714 | 0,070 | 0,000 | 0,000 | 0,024 | 0 |
| T26/P57 | 737 | 0,556 | 0,068 | 0,000 | 0,118 | 3 |
| T26/P62 | 528 | 0,000 | 0,000 | 0,000 | 0,008 | 0 |
| T26/P63 | 347 | 0,231 | 0,000 | 0,000 | 0,026 | 2 |
| T26/P66 | 892 | 0,101 | 0,000 | 0,000 | 0,030 | 0 |
| T26/P75 | 493 | 0,223 | 0,020 | 0,000 | 0,058 | 0 |
| T26/P77 | 923 | 0,444 | 0,022 | 0,000 | 0,078 | 0 |
| T26/P78 | 772 | 0,544 | 0,052 | 0,000 | 0,087 | 2 |
| T26/P80 | 613 | 1,093 | 0,832 | 0,555 | 0,406 | 13 |
| T26/P81 | 835 | 0,120 | 0,000 | 0,000 | 0,027 | 0 |
| T26/P82 | 680 | 1,074 | 0,500 | 0,162 | 0,263 | 4 |
| T26/P86 | 1451 | 1,110 | 0,448 | 0,103 | 0,220 | 0 |
| T26/P95 | 555 | 0,234 | 0,000 | 0,000 | 0,045 | 0 |
| T28/P01 | 1238 | 1,422 | 0,557 | 0,202 | 0,306 | 0 |
| T28/P02 | 1197 | 0,167 | 0,000 | 0,000 | 0,026 | 0 |
| T28/P04 | 2407 | 0,071 | 0,000 | 0,000 | 0,025 | 0 |
| T28/P05 | 113 | 0,000 | 0,000 | 0,000 | 0,000 | 0 |
| T28/P06 | 471 | 0,297 | 0,000 | 0,000 | 0,034 | 0 |
| T28/P07 | 927 | 0,043 | 0,000 | 0,000 | 0,016 | 0 |
| T28/P10 | 439 | 0,000 | 0,000 | 0,000 | 0,005 | 0 |
| T28/P11 | 590 | 0,508 | 0,034 | 0,000 | 0,065 | 0 |
| T28/P12 | 764 | 0,000 | 0,000 | 0,000 | 0,002 | 0 |
| T28/P13 | 1167 | 0,463 | 0,000 | 0,000 | 0,052 | 0 |
| T28/P14 | 1034 | 0,000 | 0,000 | 0,000 | 0,032 | 0 |
| T28/P15 | 942 | 0,011 | 0,000 | 0,000 | 0,006 | 0 |
| T28/P16 | 857 | 0,000 | 0,000 | 0,000 | 0,002 | 0 |
| T28/P17 | 553 | 0,145 | 0,018 | 0,000 | 0,032 | 0 |
| T28/P18 | 650 | 1,046 | 0,046 | 0,000 | 0,073 | 0 |
| T28/P19 | 1293 | 0,232 | 0,008 | 0,000 | 0,054 | 0 |
| T28/P20 | 1940 | 0,046 | 0,000 | 0,000 | 0,009 | 0 |
| T28/P21 | 662 | 0,000 | 0,000 | 0,000 | 0,000 | 0 |
| T28/P22 | 1480 | 0,061 | 0,000 | 0,000 | 0,034 | 0 |
| T28/P23 | 665 | 0,000 | 0,000 | 0,000 | 0,000 | 0 |
| T28/P24 | 701 | 0,043 | 0,000 | 0,000 | 0,017 | 0 |
| T28/P25 | 552 | 0,036 | 0,000 | 0,000 | 0,007 | 0 |
| T28/P26 | 760 | 1,132 | 0,000 | 0,000 | 0,083 | 0 |
| T28/P28 | 504 | 0,000 | 0,000 | 0,000 | 0,028 | 0 |
| T28/P29 | 671 | 0,015 | 0,000 | 0,000 | 0,017 | 0 |
| T28/P30 | 706 | 0,000 | 0,000 | 0,000 | 0,001 | 0 |
| T28/P31 | 1471 | 0,007 | 0,000 | 0,000 | 0,010 | 0 |
| T28/P33 | 1010 | 0,782 | 0,010 | 0,000 | 0,093 | 0 |
| T28/P34 | 632 | 0,000 | 0,000 | 0,000 | 0,002 | 0 |
| T28/P35 | 691 | 0,116 | 0,014 | 0,000 | 0,041 | 0 |
| T28/P36 | 717 | 0,112 | 0,000 | 0,000 | 0,018 | 0 |
| T28/P38 | 404 | 0,693 | 0,124 | 0,000 | 0,119 | 0 |
| T29/P01 | 545 | 0,000 | 0,000 | 0,000 | 0,004 | 0 |
| T29/P02 | 450 | 0,044 | 0,000 | 0,000 | 0,021 | 0 |
| T29/P03 | 392 | 0,408 | 0,000 | 0,000 | 0,059 | 0 |
| T29/P07 | 416 | 0,048 | 0,000 | 0,000 | 0,018 | 2 |
| T29/P08 | 508 | 0,118 | 0,000 | 0,000 | 0,023 | 0 |
| T29/P09 | 449 | 0,980 | 0,735 | 0,356 | 0,330 | 12 |
| T29/P10 | 718 | 0,014 | 0,000 | 0,000 | 0,012 | 2 |
| T29/P11 | 1097 | 0,602 | 0,000 | 0,000 | 0,056 | 0 |
| T29/P12 | 1190 | 0,000 | 0,000 | 0,000 | 0,016 | 0 |
| T29/P15 | 644 | 0,000 | 0,000 | 0,000 | 0,004 | 0 |
| T29/P17 | 503 | 0,099 | 0,000 | 0,000 | 0,024 | 0 |
| T29/P18 | 269 | 0,000 | 0,000 | 0,000 | 0,021 | 0 |
| T29/P19 | 627 | 1,085 | 0,000 | 0,000 | 0,088 | 0 |
| T29/P20 | 549 | 0,000 | 0,000 | 0,000 | 0,019 | 1 |
| T29/P21 | 928 | 0,032 | 0,000 | 0,000 | 0,010 | 0 |
| T29/P22 | 695 | 0,000 | 0,000 | 0,000 | 0,014 | 0 |
| T29/P25 | 859 | 0,000 | 0,000 | 0,000 | 0,018 | 0 |
| T29/P27 | 545 | 1,138 | 0,018 | 0,000 | 0,111 | 0 |
| T29/P28 | 1300 | 0,000 | 0,000 | 0,000 | 0,005 | 0 |
| T29/P29 | 717 | 0,181 | 0,000 | 0,000 | 0,025 | 0 |
| T29/P30 | 187 | 0,107 | 0,000 | 0,000 | 0,028 | 0 |
| T29/P31 | 458 | 0,240 | 0,000 | 0,000 | 0,048 | 1 |
| T29/P32 | 598 | 0,050 | 0,000 | 0,000 | 0,041 | 0 |
| T29/P36 | 624 | 1,442 | 1,010 | 0,481 | 0,504 | 7 |
| T29/P37 | 845 | 0,130 | 0,000 | 0,000 | 0,024 | 0 |
| T29/P38 | 659 | 0,000 | 0,000 | 0,000 | 0,007 | 0 |
| T29/P39 | 393 | 0,000 | 0,000 | 0,000 | 0,005 | 0 |
| T29/P40 | 716 | 0,000 | 0,000 | 0,000 | 0,017 | 0 |
| T29/P41 | 419 | 0,597 | 0,048 | 0,000 | 0,085 | 1 |
| T29/P45 | 990 | 0,020 | 0,000 | 0,000 | 0,018 | 0 |
| T29/P46 | 457 | 0,000 | 0,000 | 0,000 | 0,002 | 0 |
| T29/P47 | 2421 | 0,078 | 0,000 | 0,000 | 0,020 | 0 |
| T29/P48 | 516 | 0,039 | 0,000 | 0,000 | 0,017 | 0 |
| T29/P49 | 673 | 0,000 | 0,000 | 0,000 | 0,018 | 0 |
| T29/P50 | 533 | 0,000 | 0,000 | 0,000 | 0,004 | 0 |
| T32/P01 | 829 | 0,302 | 0,024 | 0,000 | 0,057 | 0 |
| T32/P02 | 822 | 0,024 | 0,000 | 0,000 | 0,012 | 0 |
| T32/P03 | 886 | 0,011 | 0,000 | 0,000 | 0,008 | 1 |
| T32/P04 | 1312 | 0,000 | 0,000 | 0,000 | 0,003 | 0 |
| T32/P05 | 558 | 0,000 | 0,000 | 0,000 | 0,023 | 0 |
| T32/P06 | 554 | 0,758 | 0,090 | 0,000 | 0,098 | 0 |
| T32/P07 | 692 | 0,867 | 0,029 | 0,000 | 0,117 | 0 |
| T32/P08 | 958 | 0,000 | 0,000 | 0,000 | 0,012 | 0 |
| T32/P09 | 864 | 0,220 | 0,000 | 0,000 | 0,037 | 0 |
| T32/P10 | 705 | 0,582 | 0,128 | 0,014 | 0,114 | 2 |
| T32/P11 | 544 | 0,037 | 0,000 | 0,000 | 0,011 | 0 |
| T32/P12 | 1082 | 2,375 | 0,028 | 0,000 | 0,182 | 0 |
| T32/P13 | 1030 | 0,903 | 0,049 | 0,000 | 0,116 | 0 |
| T32/P15 | 677 | 1,211 | 0,502 | 0,089 | 0,269 | 12 |
| T32/P17 | 331 | 0,000 | 0,000 | 0,000 | 0,019 | 0 |
| T32/P18 | 484 | 0,000 | 0,000 | 0,000 | 0,009 | 0 |
| T32/P19 | 689 | 0,015 | 0,000 | 0,000 | 0,015 | 2 |
| T32/P20 | 591 | 0,017 | 0,000 | 0,000 | 0,011 | 0 |
| T32/P21 | 624 | 0,176 | 0,000 | 0,000 | 0,036 | 0 |
| T32/P23 | 887 | 0,011 | 0,000 | 0,000 | 0,010 | 0 |
| T32/P25 | 856 | 1,624 | 0,607 | 0,210 | 0,315 | 0 |
| T32/P26 | 957 | 1,599 | 0,606 | 0,219 | 0,358 | 0 |
| T32/P27 | 614 | 0,505 | 0,000 | 0,000 | 0,083 | 0 |
| T32/P28 | 553 | 0,832 | 0,036 | 0,018 | 0,099 | 0 |
| T32/P29 | 781 | 0,909 | 0,179 | 0,038 | 0,147 | 1 |
| T32/P35 | 801 | 0,400 | 0,000 | 0,000 | 0,071 | 4 |
| T32/P36 | 496 | 0,806 | 0,222 | 0,020 | 0,165 | 0 |
| T32/P37 | 575 | 1,217 | 0,417 | 0,000 | 0,192 | 12 |
| T32/P38 | 614 | 0,342 | 0,049 | 0,000 | 0,050 | 0 |
| T32/P39 | 926 | 0,734 | 0,076 | 0,011 | 0,105 | 0 |
| T32/P45 | 889 | 0,427 | 0,000 | 0,000 | 0,068 | 0 |
| T32/P46 | 776 | 0,490 | 0,026 | 0,000 | 0,074 | 1 |
| T32/P47 | 750 | 1,200 | 0,680 | 0,027 | 0,214 | 14 |
| T32/P48 | 568 | 1,180 | 0,158 | 0,000 | 0,136 | 8 |
| T32/P49 | 584 | 1,404 | 1,027 | 0,462 | 0,400 | 20 |
| T36/P02 | 749 | 0,027 | 0,000 | 0,000 | 0,018 | 0 |
| T36/P04 | 929 | 0,065 | 0,000 | 0,000 | 0,015 | 0 |
| T36/P18 | 392 | 0,230 | 0,000 | 0,000 | 0,045 | 0 |
| T36/P24 | 966 | 0,000 | 0,000 | 0,000 | 0,004 | 0 |
| T36/P25 | 651 | 1,091 | 0,276 | 0,031 | 0,165 | 0 |
| T36/P26 | 711 | 0,942 | 0,492 | 0,014 | 0,231 | 0 |
| T36/P27 | 675 | 0,519 | 0,000 | 0,000 | 0,067 | 0 |
| T36/P28 | 664 | 1,009 | 0,196 | 0,000 | 0,134 | 0 |
| T36/P29 | 576 | 0,243 | 0,000 | 0,000 | 0,042 | 0 |
| T36/P35 | 445 | 0,180 | 0,000 | 0,000 | 0,052 | 0 |
| T36/P36 | 400 | 1,375 | 0,225 | 0,025 | 0,137 | 0 |
| T36/P37 | 405 | 0,025 | 0,000 | 0,000 | 0,015 | 0 |
| T36/P38 | 492 | 0,000 | 0,000 | 0,000 | 0,005 | 0 |
| T36/P39 | 804 | 0,174 | 0,000 | 0,000 | 0,035 | 0 |
| T36/P45 | 649 | 0,339 | 0,031 | 0,000 | 0,049 | 1 |
| T36/P46 | 533 | 0,375 | 0,000 | 0,000 | 0,066 | 0 |
| T36/P47 | 498 | 0,402 | 0,000 | 0,000 | 0,054 | 0 |
| T36/P48 | 427 | 0,164 | 0,000 | 0,000 | 0,027 | 0 |
| T36/P61 | 1047 | 0,086 | 0,010 | 0,010 | 0,028 | 0 |
| T36/P64 | 737 | 0,529 | 0,000 | 0,000 | 0,072 | 0 |
