# Supplementary figures and images for: CONS-COCOMAPS: a novel tool to measure and visualize the conservation of inter-residue contacts in multiple docking solutions
Source: BMC Bioinformatics. 2012 Mar 28;13(Suppl 4):S19. doi: 10.1186/1471-2105-13-S4-S19 (PMC3434444; doi:10.1186/1471-2105-13-S4-S19)

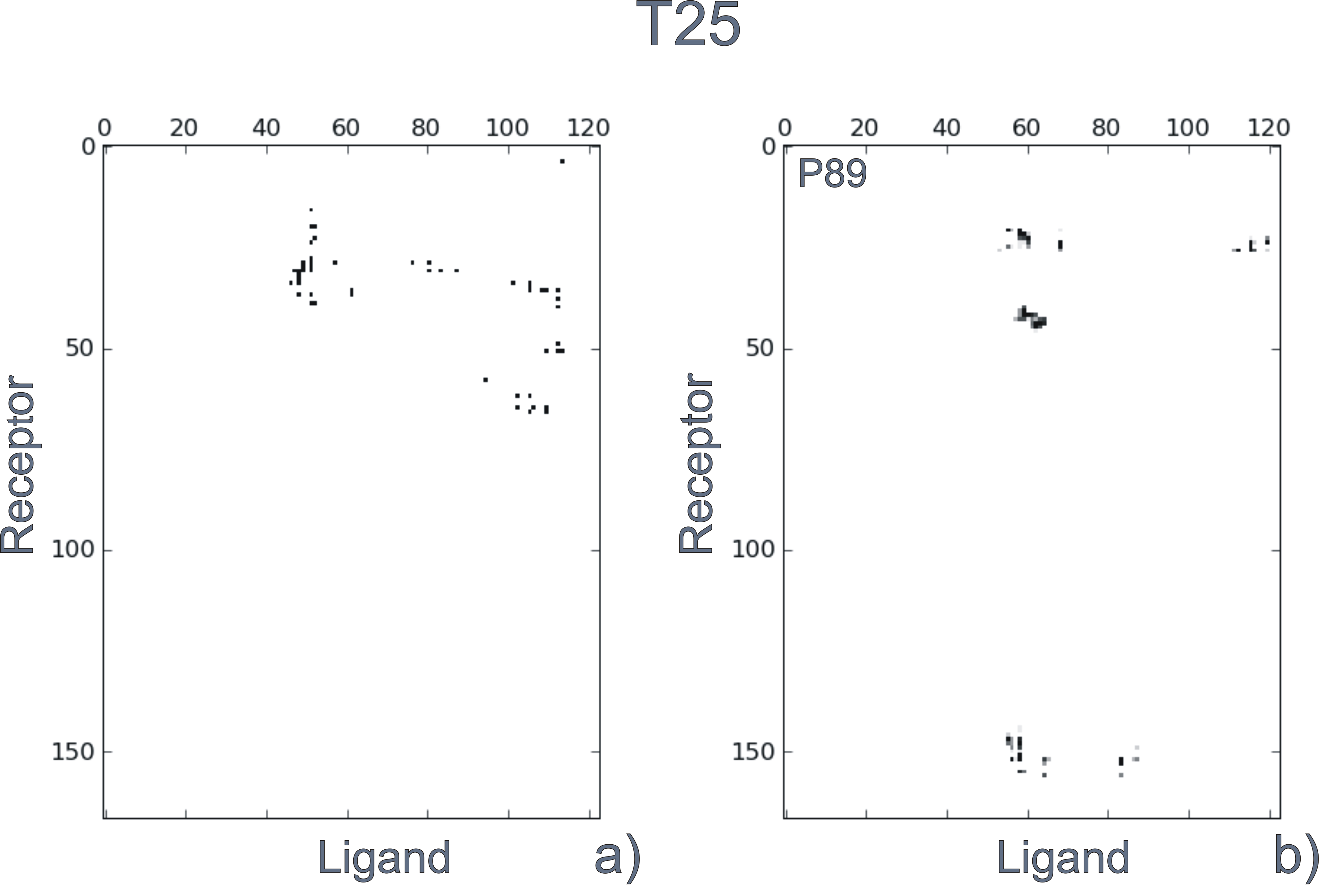

Supplement: Additional file 2 — Consensus map from the P89 predictor for T25. Comparison between the CONS-COCOMAPS consensus map (b) obtained from the 10 models submitted for the CAPRI target T25 by the P89 predictor, and the intermolecular contact map (a) of the corresponding native structure (PDB code: 2J59). [file 1471-2105-13-S4-S19-S2.tif]

T24

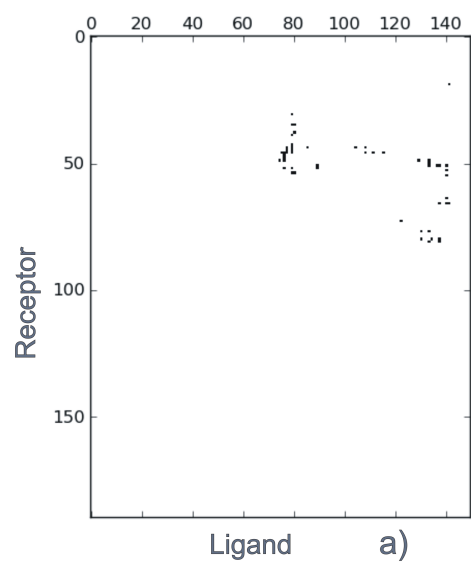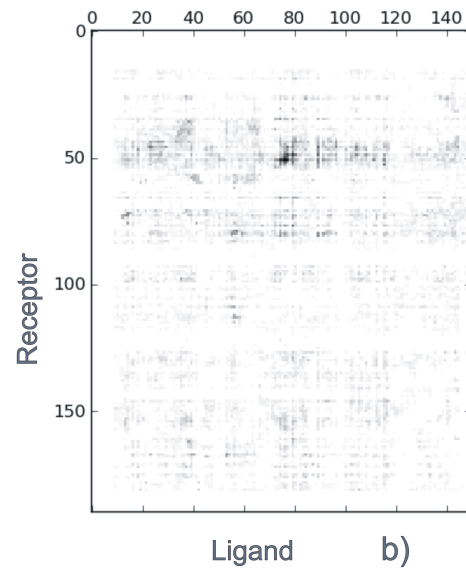

T28

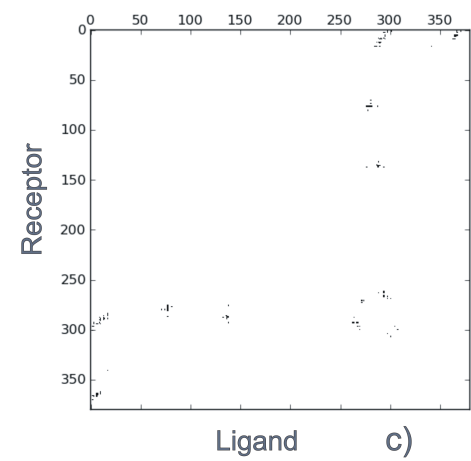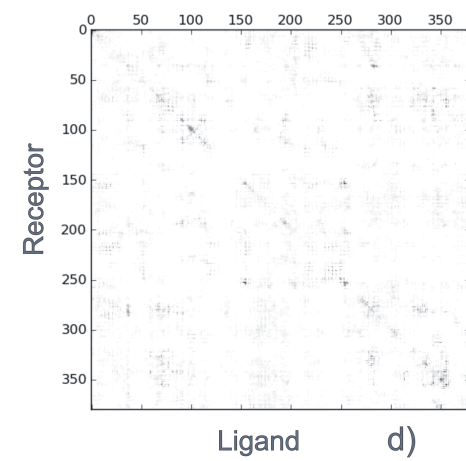

T36

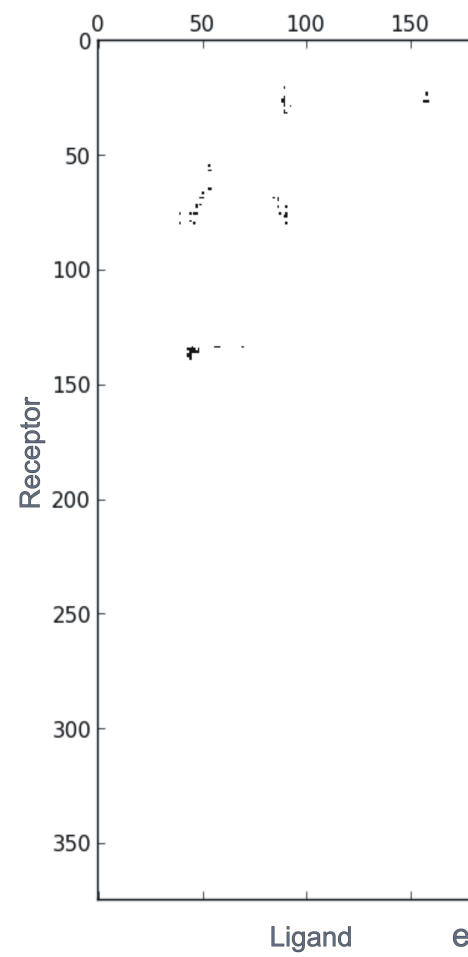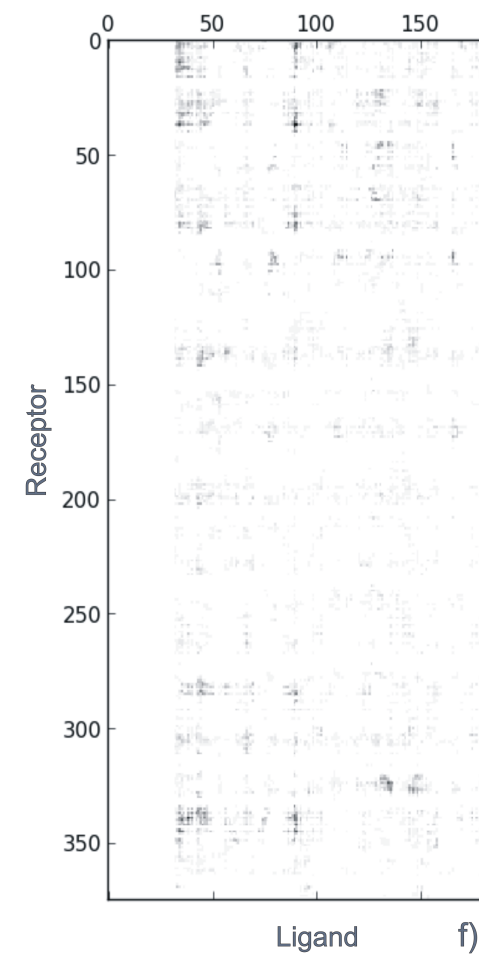

Supplement: Additional file 3 — Consensus maps for T24, T28 and T36. Comparison between the CONS-COCOMAPS consensus maps (b,d,f) obtained from all the 300, 320 and 200 models submitted to CAPRI for the targets T24, T28 and T36, respectively, and the intermolecular contact maps (a,c,e) of the corresponding native structures (PDB codes: 2J59, 2ONI and 2W5F). [file 1471-2105-13-S4-S19-S3.pdf]

T25

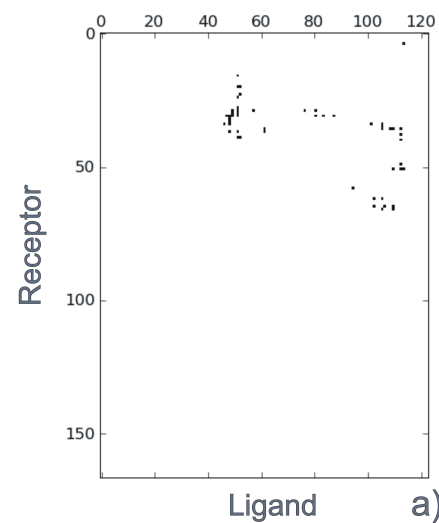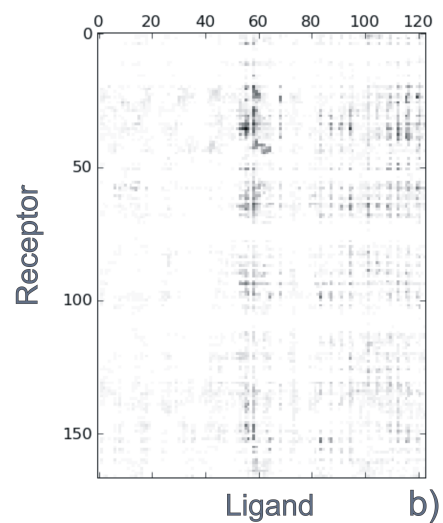

T26

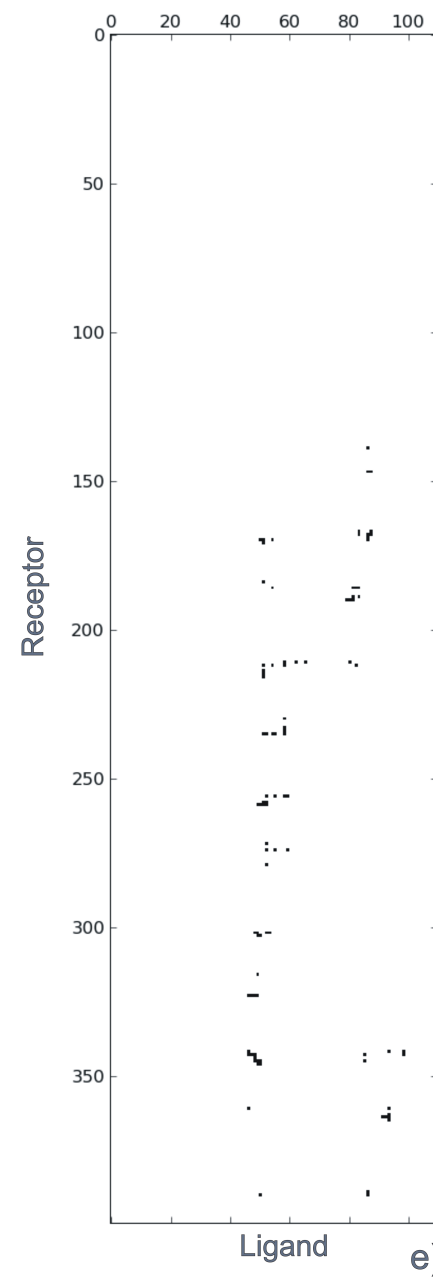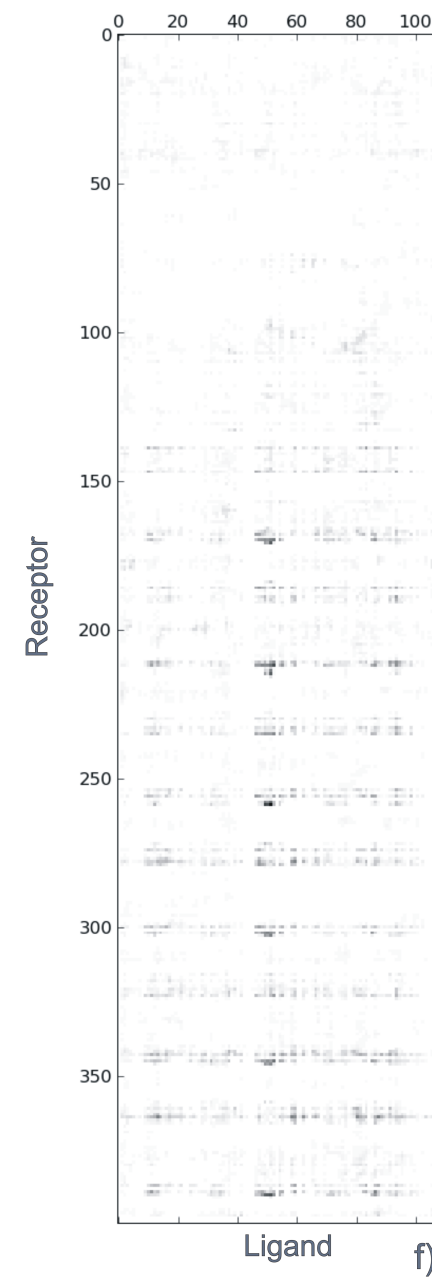

T32

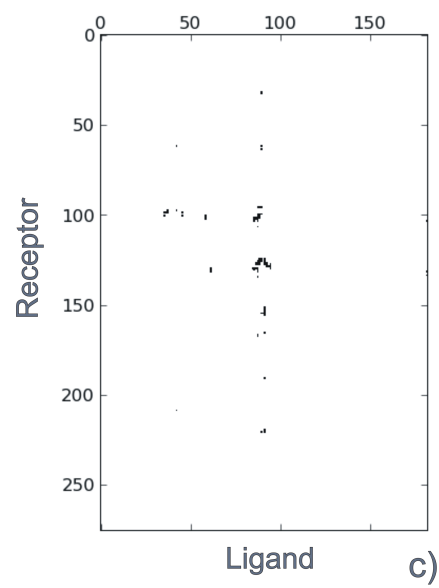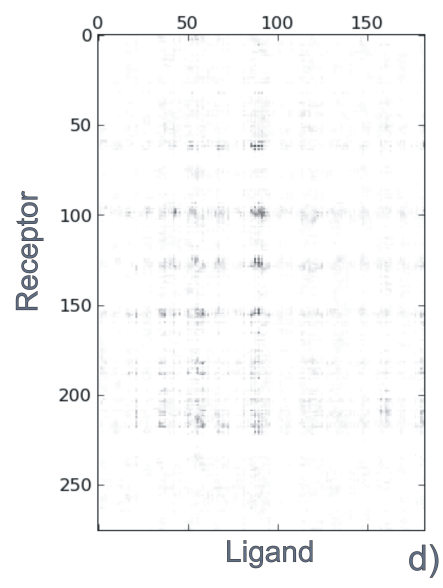

Supplement: Additional file 4 — Consensus maps for T25, T26 and T32 from incorrect models. Comparison between the CONS-COCOMAPS consensus maps (b,d,f) obtained from the 268, 276 and 316 incorrect models submitted to CAPRI for the targets T25, T26 and T32, respectively, and the intermolecular contact maps (a,c,e) of the corresponding native structures (PDB codes: 2J59, 2HQS and 3BX1). [file 1471-2105-13-S4-S19-S4.pdf]
