# Supplementary material for: CONS-COCOMAPS: a novel tool to measure and visualize the conservation of inter-residue contacts in multiple docking solutions
Source: BMC Bioinformatics. 2012 Mar 28;13(Suppl 4):S19. doi: 10.1186/1471-2105-13-S4-S19 (PMC3434444; doi:10.1186/1471-2105-13-S4-S19)
Supplement: Additional file 5 — Ten most conserved inter-residue contacts for T24 and corresponding distances in the native structure. [file 1471-2105-13-S4-S19-S5.doc]

|  | **CR_kl_** | **Receptor** | | **Ligand** | | **Distance (Å)** |
| --- | --- | --- | --- | --- | --- | --- |
| **T24** |  |  |  |  |  |  |
|  | 0,093 | PHE | 51 | ASP | 996 | 5,82 |
|  | 0,083 | PHE | 51 | ILE | 997 | 8,11 |
|  | 0,080 | PHE | 51 | LEU | 994 | 6,47 |
|  | 0,073 | PHE | 51 | ILE | 995 | 9,16 |
|  | 0,073 | ILE | 49 | TYR | 999 | 9,50 |
|  | 0,070 | ILE | 49 | ILE | 997 | 8,59 |
|  | 0,067 | GLY | 50 | ASP | 996 | 6,17 |
|  | 0,060 | ASN | 52 | ASP | 996 | 3,84 |
|  | 0,057 | ASN | 52 | TYR | 999 | 4,2 |
|  | 0,057 | ILE | 49 | ASP | 996 | 4,92 |
